# Mapping the Product Range of Interdental Brushes: Sizes, Shapes, and Forces

**DOI:** 10.3290/j.ohpd.a44035

**Published:** 2020-07-04

**Authors:** Caroline Sekundo, Hans Jörg Staehle

**Affiliations:** a Dentist, Department of Conservative Dentistry, Clinic for Oral, Dental and Maxillofacial Diseases, University Hospital Heidelberg, Heidelberg, Germany. Performed the experiments, the statistical analysis and wrote the manuscript.; b Professor, Department of Conservative Dentistry, Clinic for Oral, Dental and Maxillofacial Diseases, University Hospital Heidelberg, Heidelberg, Germany. Conception of the presented idea, supervised the project and critically revised the manuscript.

**Keywords:** dental plaque, interdental brush, oral hygiene, resistance to insertion

## Abstract

**Purpose:**

Preventive dentistry aims to improve oral hygiene, including the use of interdental cleansing aids. Clear and simple classifications may positively impact patient communication and motivate oral health behaviour. To date, there is no comparative analysis of interdental brush classifications and sizes.

**Materials and Methods:**

A total of 2320 interdental brush samples by 24 manufacturers was examined regarding their passage hole diameter (PHD) according to the ISO standard for interdental brushes (ISO16409:2016), and their current classifications were evaluated. Inter- and intrarater reliability of the ISO size classification were determined based on 20 raters and 10 interdental brushes. The insertion force for these interdental brushes was analysed in vitro.

**Results:**

Excellent intra- and interrater reliability was achieved (intraclass correlation coefficient (ICC) ≥ 0.973) overall, although greater variance was observed for bigger brush sizes. Insertion forces varied depending on size and form of the brushes, amounting to 1.58 N (SD = 1.27 N) for cylindric and tapered brushes, and to 2.31 N (SD = 0.81 N) for waist-shaped brushes. The size range of commercially available products was 0.6–5.2 mm PHD, 90% presenting with a PHD ≤ 2.0 mm. Size intervals were unsystematic. The ISO size was indicated by 33% of all manufacturers, the exact PHD by 25%.

**Conclusions:**

The determination of the PHD is a reproducible instrument for most brushes currently on the market. In vitro, forces developed based on this classification are mostly moderate, thus unlikely to cause periodontal trauma. Given the discontinuous range and unclear labelling of available products, the development of a simplified classification system by usage of the PHD may benefit the practitioner and patient alike by contributing to improve oral hygiene behaviours.

For many years, focus in dentistry has shifted from intervention to prevention, aiming to reduce risk factors for dental, gingival and periodontal diseases. Although new approaches to dental biofilm control are in development,^50^ optimising oral hygiene continues to be a central aspect of this goal. However, changing oral health behaviours towards better oral hygiene is a complex undertaking.^41^ Main approaches to administer oral healthcare advice are group interventions (ie, in schools) and, above all, one-to one sessions conducted by a dental healthcare professional (individual prophylaxis). Clear and simple language and classifications of recommended hygiene products are vital so that patients can understand relevant information. Patient empowerment affects learning and increases the probability of favourably changing the individual’s behaviour.^30,33,34^ It is therefore important to employ classifications that are easy to understand and easy to use.

Implementing oral hygiene is largely focused on the removal of plaque, as its influence on caries and periodontal disease has long been studied.^3,27,44,46,49^ The cleaning of the interdental space has been regarded as particularly critical because it is insufficiently reached by conventional tooth brushing alone.^14,25,26^ For this purpose, diverse cleaning devices are available, among them dental floss, tooth picks and interdental brushes. Although some studies have accorded a positive effect to the use of dental floss,^9,29,53^ others have shown insufficient evidence as to its benefit.^5,37^ Likewise, tooth picks do not ensure adequate plaque reduction.^18^ In comparison, interdental brushes demonstrate the greater cleansing effect.^17,22,35,36,39,40,45^ However, there are deficits regarding adequate usage and correct choice of size. At present, there is no scientific consensus concerning these questions.^11^

Any unclarity in the specification and description of interdental brushes might therefore lead to ineffective and inefficient use of interdental brushes (non-usage, overusage or wrong usage, ie, usage of brushes which either cause trauma or do not yield optimal oral hygiene). It must also be emphasised that this is not just a common case of information asymmetry between patient and healthcare professional: not only dental laypersons, but also dental teams are often overburdened with the size differentiation. In practice, numerous interdental brushes, at times with measuring probes, are tested on the patient.^7^ Unclear and fragmented product pallets impede a targeted approach.

To this end, the international guideline of the International Organization for Standardization (ISO) 16409:2016 was created. Besides evaluation of the filament and stem retention and durability, it also determines the interdental brush size. For this purpose, the smallest hole through which a brush can pass without bending is defined.^21^ This passage hole diameter (PHD in mm) is determined by a standardised measuring plate, through which the brushes are passed in descending hole diameter size with ‘clinically relevant force’.^20^ Examples of the PHD determination are depicted in Figure 1. Several PHD sizes are then joined for one ISO size. Thereby, the ISO sizes 1–3 contain 2 PHD sizes respectively, sizes 4–5 contain 3 PHD sizes, and sizes 6–7 contain 5 PHD sizes. All interdental brushes ≥ a PHD of 2.9 are classified as ISO size 8.

**Fig 1:**
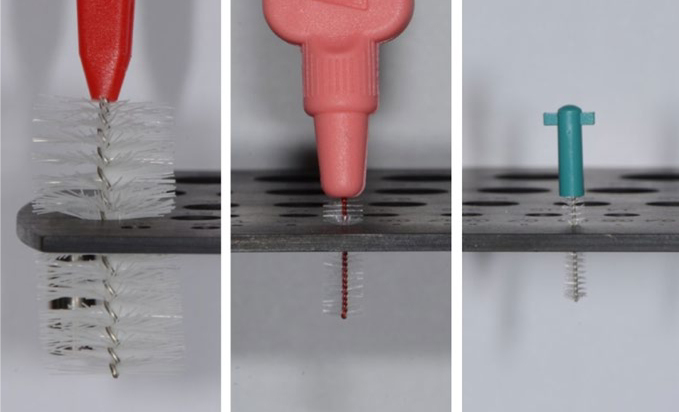
Measuring plate for PHD determination with inserted interdental brushes. From left to right: IDBG-R, TopCaredent, PHD = 5.2 mm; TePe Interdental Brush Extra Soft Red, TePe, PHD = 0.9 mm; CPS 06 prime, Curaden, PHD = 0.6 mm.

Aside from the diameter, the order, form, density, length and stiffness of the individual components can also play a role in the PHD. Currently, cylindric, tapered and waist-shaped brushes are available on the market. There is little knowledge on the relevance of these features.

An electronic search via Ovid Technologies was carried out in the Medical Literature Analysis and Retrieval System Online (MEDLINE) databank in October 2018 to identify studies evaluating different types of interdental brushes or the present ISO standard (see Appendix Table A.1). Reference lists of these studies were also searched, without leading to further results. In total, seven studies on brush form or filament type and one study relating to the ISO standard were identified. The latter, however, analysed stem durability analogous to the ISO standard, and did not determine the PHD.^19^

Among the heterogenous study goals, the electronic search showed that plaque elimination did depend on the brush diameter.^51^ In the so far only study of waist-shaped interdental brushes on eight patients, these appeared superior to the cylindrical shape.^10^ Rosing et al,^35^ as well as Bock et al,^6^ when respectively examining 50 and 110 patients, concluded that tapered and cylindrical interdental brushes have a comparable cleansing effect. The same overall outcome was observed by Larsen et al,^23^ although they concluded that cylindrical brushes cleaned the lingual surfaces better. Last but not least, Wolff et al showed in a comparison of brushes with round and triangular profiles that both eliminated the same amount of plaque, the necessary insertion forces differed, though.^52^

Against this background of inadequate data availability and low evidence concerning advantages and disadvantages of different brush forms, sizes, classifications or application forces, it is not surprising that despite existing standard, the ISO classification is not widespread. Instead, numerous rankings are in place. This lack of transparency impedes an adequate choice, denies users the possibility of comparing (and eventually changing) the manufacturer, and underlines the necessity of further research.

Despite the uncertainties concerning ‘clinically relevant force’ presented in the ISO standard, our study hypothesis is that the PHD is a reproducible instrument beneficial to size classifications of interdental brushes. Therefore, our study pursues the following objectives: (1) analysing the reliability of the ISO standard 16409:2016 for interdental brush sizes; (2) creating an overview of existing classifications and determining PHD sizes available in the oral healthcare market; and (3) determining in-vitro insertion forces among different brush forms based on the ISO classification.

## Materials and Methods

### Reliability Analysis

To evaluate the intra- and interrater reliability of the determined PHD described by the ISO standard, 10 interdental brushes (CPS 06, 07, 08, 09, 10, 11, 12, 14, 15 and 18; Curaprox/CH-Kriens) were chosen. Their PHD size was evaluated by 20 raters in a randomised order. Six dentists and 14 medical students were calibrated in one-to-one sessions, whereby the content of the ISO standard was conveyed and the stated application was demonstrated. Accordingly, the measuring plate had a thickness of 2.0 ± 0.1 mm and contained holes in 0.1 mm steps, through which eight samples of every brush were inserted in descending order. The test was terminated when reaching the smallest hole through which the sample passed completely without deformation with aforementioned ‘clinically relevant force’.^20^ For tapered brushes, an additional, smaller PHD was defined, the brush passing at least 1 mm further than the measuring plate surface, as also described by the ISO standard. This resulted in a PHD range for every tapered brush. After 1 month, raters were asked to repeat their assessments.

### Current Classifications and PHD Ranges

In the second step, size classifications currently in place by 24 manufacturers were recorded. Based on the high reliability of the ISO standard, one calibrated rater performed the PHD measurements for a total of 290 of these manufacturers’ interdental brushes (ie, eight samples each, resulting in a total of 2320 brushes tested). The number of samples per brush was chosen as required by the ISO standard. Measurement procedures were conducted as described in the previous section, ‘Reliability Analysis’.

### Insertion Forces

In a final step, the occurring force during the hole passage of the interdental brushes mentioned in the earlier section ‘Reliability Analysis’ was determined. The median of the overall 40 individual measurements was chosen as the correct hole diameter. The analysis was performed using the Zwick/Roell test machine Z005 with a 5 N force transducer and testXpert II software. A total of 13 samples of each interdental brush were tested. The first three samples were measured with varying testing speed to quantify their influence. After 10 setting cycles, the samples were measured with 1 mm/s, 3 mm/s and 6 mm/s for 20 cycles. To simulate the in-vivo passage speed, the following 10 samples were measured with the highest speed of 6 mm/s for 20 cycles. The test distance corresponded to the length of the respective interdental brush head. As different brush forms (cylindrical, tapered) were reflected in the force profile, 10 samples each of four additional interdental brush types (Circum, Top Caredent) with a waist-shaped profile were examined (sizes 2, 4, 5 and 6).

### Statistical Analysis

SPSS statistics software for Microsoft (Microsoft; Seattle, WA, USA) was used to analyse the data. The intra- and interrater reliability was assessed using the intraclass correlation coefficient (ICC) for absolute agreement. Values above 0.75 were rated as good clinical reliability.^13,32,43^ As the data was not normally distributed, Spearman rank correlation analyses measured bivariate correlation. Interpretation of correlation coefficients was based on Bühl.^8^ P values < 0.05 were considered statistically significant.

## Results

### Reliability Analysis

High intra- and interrater reliability was achieved (Table 1). The intrarater reliability was between 0.954 and 0.998 (ICC). The comparison of dentists and medical students showed a minimal difference in the choice of PHD. On average, it was 0.03 mm (1/3 PHD size) higher among the dental examiners (SD = 0.04, 95%CI: 0.02; 0.05). The average span of rater measurements was 0.31 mm (SD = 0.16, 95%CI: 0.21; 0.41). Overall, the span varied between 0.1 mm and 0.7 mm depending on the interdental brush (1–7 PHD sizes). Spearman rank correlation resulted in a moderate positive relationship between the span and size of the interdental brush (rs (12) = 0.695, p < 0.001), ie, the bigger the brush, the greater the differences between raters’ choice in PHD size. Table 2 shows an overview of measured values.

**Table 1:** Intra- and interrater-reliability (n = 20)

	ICC (3.1)	95% confidence interval		F-test with true value 0			
		lower bound	upper bound	value	df1	df2	Sig. (p)
Interrater reliability 1. observation time (0 M)	0.976	0.951	0.992	1121.13	11	209	< 0.001
Interrater reliability 2. observation time (1 M)	0.973	0.945	0.991	937.45	11	209	< 0.001
Interrater reliability 1.+2. observation time	0.973	0.955	0.987	950.67	23	437	< 0.001
Intrarater reliability: Mean ±SD	0.9850 ± 0.012	0.928 ± 0.099	0.996 ± 0.0031	349.51 ± 364.96	11	11	< 0.001

**Table 2:** PHD measurements by 20 raters

	Median	Mean	Standard deviation (SD)	Min	Max	95% Confidence interval	
						lower bound	upper bound
CPS 06 prime	0.6	0.62	0.04	0.6	0.7	0.61	0.63
CPS 07 prime	0.7	0.73	0.06	0.6	0.9	0.71	0.75
CPS 08 prime	0.9	0.86	0.05	0.8	1.0	0.84	0.88
CPS 09 prime	0.9	0.91	0.06	0.8	1.1	0.89	0.93
CPS 10 regular	0.9	0.86	0.06	0.8	1.0	0.84	0.88
CPS 11 prime	1.1	1.06	0.07	0.9	1.2	1.04	1.08
CPS 12 prime	1.2	1.20	0.08	1.1	1.3	1.17	1.22
CPS 14 regular (tapered) min	1.3	1.27	0.05	1.1	1.3	1.25	1.28
max	1.5	1.47	0.09	1.3	1.7	1.44	1.50
CPS 15 regular (tapered) min	1.3	1.34	0.07	1.2	1.5	1.31	1.36
max	1.6	1.55	0.10	1.3	1.8	1.52	1.58
CPS 18 regular	2.5	2.54	0.17	2.3	3.0	2.48	2.59

### Current Classifications and PHD Ranges

For an overview of all manufacturers and their specifications relating to the size of interdental brushes, see Table A.2 in the Appendix. Next to the ubiquitous colour-coding which was specific to each supplier, the diameter of the brush was the most often stated information (79.2%). This was followed by the wire diameter (45.8%). A third of manufacturers stated the ISO size, and only 25.0% indicated the PHD.

**Fig 2:**
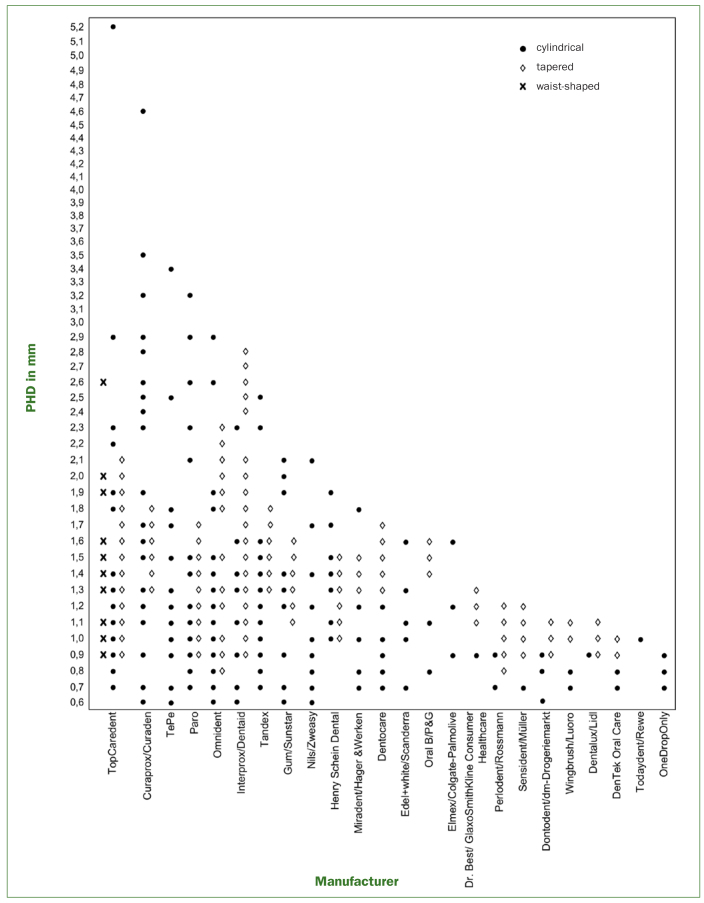
PHD sizes currently available.

The PHD determination of the 290 interdental brushes by the listed manufacturers (see Fig 2) showed a concentration on smaller sized interdental brushes. Sizes ranged between 0.6 mm and 5.2 mm. At 10.5%, the most frequent size was 0.9 mm, followed by a PHD of 1.1 mm with 8.8%. A total of 75% of all interdental brushes had a PHD ≤ 1.5 mm, only 10.0% of all brushes had a PHD > 2.0 mm. Some 77.9% of all interdental aids were cylindrical brushes, 17.9% tapered and 4.1% waist shaped. Only one manufacturer (Top Caredent) supplied waist-shaped interdental brushes, 18 supplied tapered brushes, while cylindrical shapes were produced by all. On average, tapered brushes covered a range of 3.3 ± 1.1 PHD. The range was dependent on length and conicity and was 2–6 sizes. With 39.2% most tapered brushes covered three sizes, followed by 25.5% which covered two sizes (for a complete overview of products and their PHD range, see Appendix Table A.3).

Except for a few manufacturers with a small product range (eg, DenTek Oral Care, Maryville, USA; OneDropOnly, D-Berlin) the product choice is discontinuous. Figure 2 shows an overview of currently available PHD sizes. For tapered brushes, all PHD sizes covered are marked.

### Insertion Forces

**Fig 3:**
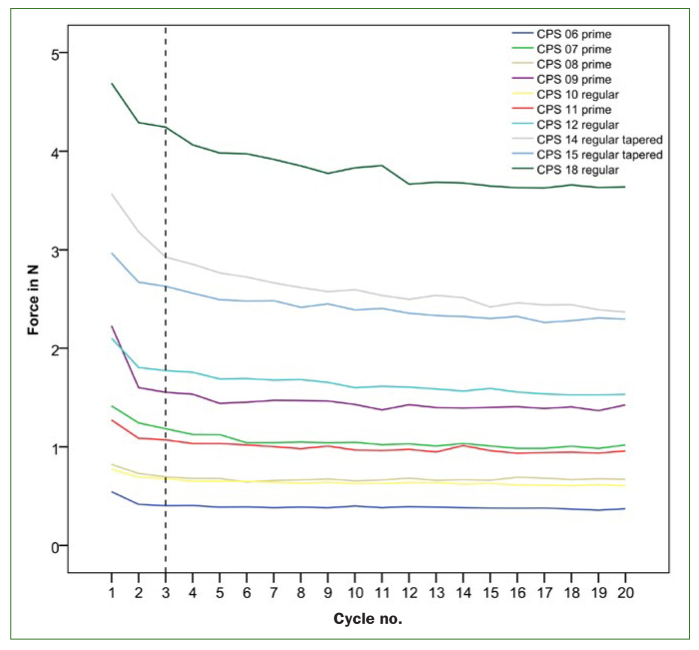
Maximum force (mean values from 10 samples).

The in-vitro test speed analysis of the rated Curaprox interdental brushes showed a decrease in insertion force with increasing speed. An increase from 1 mm/s to 6 mm/s resulted in an average reduction of 0.18 N (SD = 0.13 N). However, this difference was not statistically significant (Spearman rank correlation; p = 0.3). Therefore, the following 10 samples were measured with 6 mm/s, coming closest to the speed applied in-vivo (Figure 3). The average maximal insertion forces were between 0.41 N (CPS prime 06) and 3.83 N (CPS 18) (average values from 10 samples/20 cycles). The mean was 1.58 N (SD = 1.27). High positive correlation was present between the insertion force and the determined PHD size, ie, the bigger the interdental brush, the bigger the necessary insertion force (rs (10) = 0.853; p < 0.02) for the PHD chosen.

The force decreased with growing cycle number, the biggest force reduction took place in the first three cycles (see dotted line, Fig 3). In these first cycles, the force decreased by 0.32 N (SD = 0.21 N). This is equivalent to 17.1% (SD = 6.46%) of the original value achieved on first insertion. After this initial force reduction, the mean force was between 0.41 N (CPS prime 06) and 3.64 N (CPS 18). The size of the brush (the PHD) and the duration of negative force development (cycle no.) showed a high positive correlation (rs (8) = 0.784, p < 0.007). Hence, with increasing interdental brush size, the number of applications until the brush reaches a constant force level during insertion and removal is greater. The mean total force reduction was 0.50 N respectively 23.5% (SD = 7.51%). A linear regression model was used to quantify the role of maximal insertion force as a predictor of force reduction. The outcome showed a statistically significant regression equation (F (1, 8) = 13.298; p = 0.007) with a R2 of 0.624. The insertion force fell by 0.264 N for every N of initial maximal insertion force.

**Fig 4:**
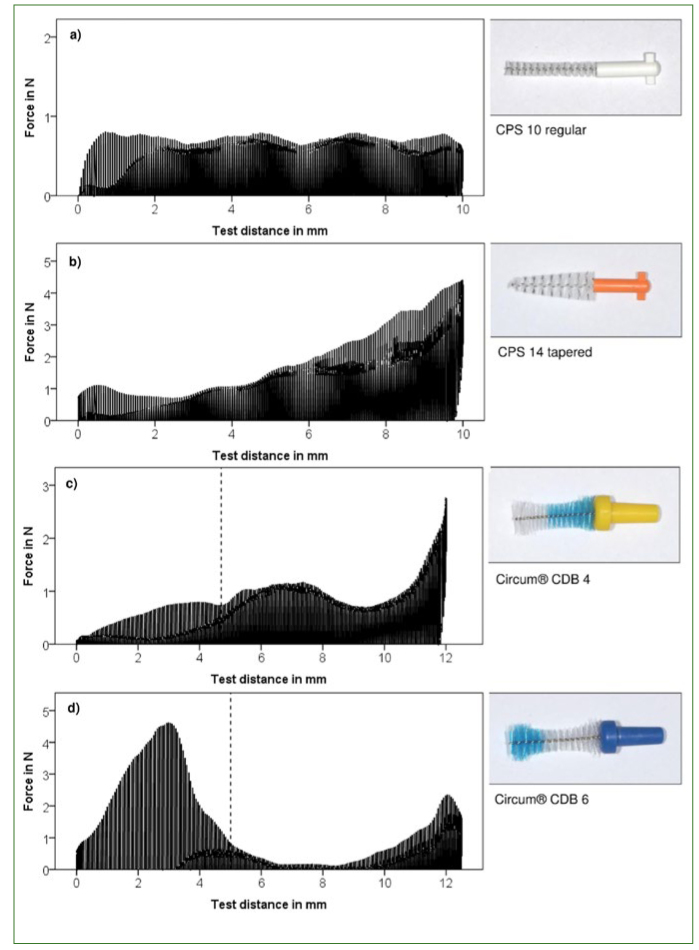
Exemplary illustration of the force profile for one of each brush type examined: superimposition of 20 cycles. (a) Cylindrical, (b) tapered, (c) and (d) waist shaped.

The following analysis of Circum interdental brushes with waist-shaped profile yielded in maximal force between 1.60 N and 3.82 N depending on the brush size (average of 10 samples) and an overall mean of 2.31 N (SD = 0.81 N). The force progression during hole passage proved to be dependent on form and bristle type of the interdental brush (shown exemplary in Fig 4). The presented Circum interdental brush included a change in bristle type after approximately one-third of the brush head. Whereas the Circum sizes 2 and 4 had a softer bristle type at the tip and stiffer bristles towards the shaft, sizes 5 and 6 were constructed in reverse order. This resulted in higher insertion forces towards the end or the beginning of the hole passage, respectively, reaching values between 4.74 N and 6.78 N. This change in force becomes apparent in Figure 4 (c and d) (dotted line). After this initial change, the force level fell in the waist area of the brush, and then steeply increased with a mean gradient of 0.96 N/mm.

A constant force level was achieved with consistent bristle type and cylindrical brush form, as is shown here by way of example with the CPS 10 regular in Figure 4a. The variance of the insertion force was low and amounted to 0.007N2. The range was 0.25 N and was based on the switch from one bristle bundle to another. Tapered brushes showed a consistent increase in insertion force. At a conicity of 20 degrees, the force increased by 0.60 N per mm test distance towards the brush shaft.

## Discussion

There is insufficient evidence of correct choice and sizing of interdental brushes despite the importance of supplementary interdental cleaning^12^ and the relevance of interdental brushes.^17,22,35,36,39,40,45^ This is particularly disadvantageous when aiming to change people’s oral health behaviour towards better oral hygiene.

The evaluation of the ISO standard 16409:2016 showed that, regardless of unclear instruction concerning the required force to determine the PHD, excellent intra- and interrater reliability was achieved. More reliable results were observed with smaller-sized brushes. Because of statistically higher spans in sizing of bigger brushes, their classification must be viewed critically. However, at the current market offerings, this plays a minor role as 90% of interdental brushes have a PHD ≤ 2.0 mm.

The generally reduced offerings of interdental brushes with a PHD above 2.0 mm could be because the handling of smaller sizes is perceived as more comfortable. Possibly, manufacturers also target the greater client group with no or minimal periodontal disease. However, especially patients with pronounced periodontitis need a balanced offer of bigger-sized interdental brushes. A continuous product range would therefore be recommendable.

Current product classification and labelling systems appear to be highly diverse and may complicate the uptake and continuous use of interdental brushes by patients. The most commonly used parameters, brush and wire diameter, can only provide limited information since the passage is also dependent on form, bristle type and arrangement, and different diameter brushes can pass through the same interdental space.^42^ Subsequently, only the PHD value is of interest to dentist and patient alike. At present, only a quarter of all manufacturers declare the PHD. Therefore, a wider distribution is desirable. Nonetheless, it must be questioned whether the joining of PHD sizes to ISO sizes has a practical advantage. Clinical observations have shown that an interdental space cannot be cleaned equally efficiently by all sizes contained in an ISO nomenclature, as smaller interdental brushes result in inferior subgingival cleansing.^42^ Therefore, a dismissal of the ISO nomenclature in favour of PHD labelling might be of value.

Classifications should also warrant safe use. Therefore, the question to be asked is: ‘Which force spectrum should be considered acceptable for intraoral application?’ Our results have shown that the subjective ‘clinically relevant’ force described by ISO nomenclature is not constant over all brush sizes. Rater chose a PHD with greater necessary insertion force for bigger brushes. Even though this study shows faster decrease in force for brushes with higher initial insertion force, hard and soft tissue trauma caused in the initial phase of each new interdental brush cannot be ruled out.

Gingival and dental trauma as a side effect of tooth brushes and dental floss has been described for a long time.^1,2,16,31,38,54^ Vogel et al also observed this effect by interdental brushes, stating that injury of the gingiva is dependent on the duration of cleaning and type of interdental brush but no evaluation of the applied force took place.^47^ However, the force also correlates with arising trauma.^4,28,48^ On the basis of these studies on conventional tooth brushing, an adequate force of 3N was proposed in order to prevent damage of hard and soft tissues.^15^

If one assumes this value for interdental trauma, the majority of examined interdental brushes are, on average, in the safe spectrum with 1.58 ± 1.27 N. It was surpassed by the biggest interdental brush (PHD = 2.5). Likewise, the tapered and waist-shaped interdental brushes of middle size surpassed the 3 N limit. Ultimately, the intended benefit must be balanced against potential disadvantages (eg, compression of the gingival papilla or gingival recessions^24,28^). This takes place against the background of unclear advantages of these brush forms in plaque elimination.^6,23,35^ However, due to the limitations of this study as an in-vitro analysis, the consequences of the insertion forces measured cannot be foreseen. Future in-vivo studies regarding traumatising side effects of different interdental brush types must take place to allow for a concluding weighing of advantages and disadvantages.

## Conclusion

In summary, given the widely dispersed range of available products and scattered distribution of products alongside the range of potentially required PHDs, oral healthcare professionals and patients may not always find it straightforward to identify optimal interdental brushes. The PHD determination according to ISO 16409:2016 presents a reliable instrument for size classification for the majority of currently available interdental brushes. In vitro, these interdental brushes develop moderate forces, with few exceptions. Very big, tapered and waist-shaped brushes can develop higher insertion forces based on this sizing method, which does not rule out potential damage of the periodontal apparatus. Nonetheless, bigger sizes are especially required for certain patient groups. The development of a simplified classification and labelling system for interdental brushes based on the PHD might contribute to further improve oral hygiene behaviours and hence people’s oral health.

## Appendix

**Table A.1:** Ovid Search in the Medical Literature Analysis and Retrieval System Online (MEDLINE) database (07.10.18 15:34 CET)

1. exp DENTISTRY/
2. exp Oral Hygiene/or exp Dental Plaque/
3. exp Periodontal Diseases/
4. 1 or 2 or 3
5. (interdental adj1 brush*).tw.
6. (interproximal adj1 brush*).tw.
7. 5 or 6
8. 4 and 7
9. angle*.tw.
10. conic*.tw.
11. round*.tw.
12. straight*.tw.
13. taper*.tw.
14. waist-shape*.tw.
15. triangul*.tw.
16. cylindric*.tw.
17. 9 or 10 or 11 or 12 or 13 or 14 or 15 or 16
18. ISO.tw.
19. (International adj1 Organization adj2 Standardization).tw.
20. 18 or 19
21. 7 and 20
22. 8 and 17
23. 8 or 21 or 22

**Table A.2:** Overview of interdental brush classifications in use

Product name, brand/manufacturer, country, city	ISO size	PHD*	Wire Ø	Brush Ø	Colour coding
Circum/IDBH/IDBG/IDB, TopCaredent, CH-Zurich		X		X	X
Curaprox/Curaden, CH-Kriens		X**		X	X
TePe Interdental brush Angle/Original/X-Soft, TePe, SE-Malmö	X	X	X		X
Paro, CH-Zurich			X	X	X
Omnident Interdental brush with handle/Interdental brush for holder/Interdental brush, Omnident, D-Rodgau		X		X	X
Interprox/Dentaid, D-Mannheim	X	X	X	X	X
FLEXI/FLEXI Ultrasoft/FLEXImax/TANDEX PROXI/TANDEX CLASSIC, Tandex, DK-Lynge	X		X	X	X
Trav-Ler/Bi-Direction, Gum/Sunstar, CH-Etoy	X	X	X		X
Nils/Zweasy, D-Trier				X	X
Interdental brush Acclean/Interdental Travel brushes Acclean, Henry Schein Dental, D-Langen				X	X
Pic-Brush, Miradent/Hager &Werken, D-Duisburg			X	X	X
Proximal Grip Classic, Dentocare, D-Höhenkirchen			X		X
Easy Flex, Edel+white/Scanderra, CH-Zurich			X	X	X
Interdental brush Oral B/P&G, D-Schwalbach am Taunus				X	X
Interdental brush Elmex/Colgate-Palmolive USA, New York				X	X
Interdental brush Dr. Best/GlaxoSmithKline Consumer Healthcare, D-Munich				X	X
Interdental brush, Perlodent med/Rossmann. D-Burgwedel	X			X	X
Interdental Stick, Sensident/Müller, D-Ulm-Jungingen	X			X	X
Interdental Sticks, Dontodent/dm-Drogeriemarkt, D-Karlsruhe	X		X	X	X
Wingbrush/Luoro, D-Cologne	X		X	X	X
Interdent brush, Dentalux/Lidl, D-Neckarsulm				X	X
Easy Brush/Slim Brush, DenTek Oral Care, Maryville, USA				X	X
Today dent/Rewe, D-Cologne					X
Interdental brush OneDropOnly, D-Berlin			X		X

* or similar, eg, ‘easy application size’; ** limited validity, for big sizes generically >2.0 mm.

**Table A.3:** PHD range of tapered interdental brushes

No. of covered PHD sizes	No. of products	Product name, brand/manufacturer
2	13	Easy Brush fine, DenTek Oral Care; Isola F #1049, Paro; Flexigrip orange x-fine, Paro; Tapered Fine Interdental Brush, Perlodent med; Trav-Ler 1414, Gum/Sunstar; ISO 3 Wingbrush, Luoro; ISO 3 Interdental Stick, Sensident; Tapered Fine Interdental Stick, Sensident; ISO 3 brushes for refill, Dontodent; IDBH-GK, TopCaredent; 1026324 Interdental Travel Brushes Acclean, Henry Schein; Interdental Brush Green-Tapered Fine, Interdental Brush with handle blue tapered, Omnident
3	20	Tapered Fine Interdental Brush, Dr. Best; Isola Long #1010, Paro; Isola F #1046, Paro; Tapered Fine Interdental Brush for refill, Perlodent med; Pic-Brush conical, Miradent; Bi-Direction rosa, Gum/Sunstar; Trav-Ler 1614, Gum/Sunstar; CPS regular 14Z, Curaprox/Curaden; CPS regular 15, Curaprox/Curaden; interprox mini conical, Dentaid; Interprox plus Miniconical, Dentaid; interprox plus conical, Dentaid; TANDEX PROXI x-fine tapered, Tandex; ISO 3 Interdental Sticks, Dontodent; IDBH-RK, IDBG-GK, IDB-GK, TopCaredent; Tapered Fine Interdental Brush, Dentalux; 9002456 Interdental Brushes Acclean, Henry Schein; Interdental Brush Tapered, Oral B
4	8	Isola Long #1011, Paro; CPS 25 strong & implant, Curaprox/Curaden; Interprox Conical, Dentaid; FLEXI Ultrasoft dark grey, Tandex; FLEXImax Purple, Tandex; 1028793 Interdental Travel Brushes Acclean, Henry Schein; Interdental Brush Green-Tapered X-Fine, Interdental Brush Red Tapered, Omnident
5	8	12840 Proximal Grip Classic blue, Dentocare; CPS 508 Soft Implant, Curaprox/Curaden; LS 634, Curaprox/Curaden; interprox plus XX-maxi, Dentaid; FLEXI Lilac, Tandex; FLEXI Lime, Tandex; IDBG-VK, IDB-VK, Top Caredent
6	2	Interprox Plus X-maxi, Dentaid; Interdental Brush Violet Tapered, Omnident
